# Regulation of the Actin Cytoskeleton in Podocytes

**DOI:** 10.3390/cells9071700

**Published:** 2020-07-16

**Authors:** Judith Blaine, James Dylewski

**Affiliations:** 1 Renal Division, University of Colorado Anschutz Medical Campus, Aurora, CO 80045, USA; judith.blaine@cuanschutz.edu; 2 Renal Division, University of Colorado Anschutz Medical Campus and Denver Health Medical Center, Aurora, CO 80045, USA

**Keywords:** podocyte, actin cytoskeleton, foot process, slit diaphragm, focal adhesion

## Abstract

Podocytes are an integral part of the glomerular filtration barrier, a structure that prevents filtration of large proteins and macromolecules into the urine. Podocyte function is dependent on actin cytoskeleton regulation within the foot processes, structures that link podocytes to the glomerular basement membrane. Actin cytoskeleton dynamics in podocyte foot processes are complex and regulated by multiple proteins and other factors. There are two key signal integration and structural hubs within foot processes that regulate the actin cytoskeleton: the slit diaphragm and focal adhesions. Both modulate actin filament extension as well as foot process mobility. No matter what the initial cause, the final common pathway of podocyte damage is dysregulation of the actin cytoskeleton leading to foot process retraction and proteinuria. Disruption of the actin cytoskeleton can be due to acquired causes or to genetic mutations in key actin regulatory and signaling proteins. Here, we describe the major structural and signaling components that regulate the actin cytoskeleton in podocytes as well as acquired and genetic causes of actin dysregulation.

## 1. Introduction

Proteinuria is one of the hallmarks of kidney disease. Under normal circumstances, less than 150 mg a day of protein is found in the urine. Leakage of serum proteins into the urine is prevented by the glomerular filtration barrier (GFB), a structure consisting of fenestrated endothelial cells, the glomerular basement membrane (GBM) and podocytes. Podocytes are terminally differentiated cells that form the outer layer of the GFB and are exposed to significant mechanical forces: tensile stress and fluid flow shear stress [1]. Tensile stress is generated by changes in glomerular capillary pressure which stretches podocyte foot processes. Shear stress is generated by the flow of glomerular ultrafiltrate which generates stress on the slit diaphragm, major processes and cell body [2]. Podocytes respond to changes in hemodynamic forces within the glomerulus by modifying the foot process actin cytoskeleton. Maintenance of the actin cytoskeleton and actin cytoskeletal dynamics are thus critical for normal podocyte function and preservation of the integrity of the glomerular filtration barrier. Podocyte actin dynamics are controlled by two structural elements within the podocyte, the slit diaphragm (SD) and focal adhesions (FAs), as well as other mediators such as cytokines and drugs. Actin cytoskeleton dysfunction in podocytes can be caused by both acquired and genetic factors. Below, we review the major structural components that regulate the actin cytoskeleton in podocytes and genetic and acquired factors that can modulate actin cytoskeleton dynamics. Before addressing the actin cytoskeleton in podocytes in particular, we briefly review the mechanisms underlying actin cytoskeleton regulation.

## 2. Structural Regulation of Actin Dynamics in Podocytes

### 2.1. Actin Dynamics

Changes in the actin cytoskeleton within a cell are necessary for maintenance of cell shape, cell motility and intracellular transport [3]. To carry out these functions, actin filaments are arranged in a variety of ways. The building blocks of actin filaments are 42 kDa actin monomers [4]. Filaments are extended by nucleation, or the formation of actin dimers and trimers, which is the rate limiting step for filament extension [5]. As soon as actin trimers are formed, they are rapidly added to the growing actin filament. Actin filaments are polar structures with two different ends: a barbed end which is highly dynamic and elongates rapidly and a pointed end which elongates at a rate ten-fold slower than that of the barbed end [6]. At the front of the cell, actin filaments are arranged into branched and cross-linked structures known as lamellipodia. Filopodia are protrusions of aligned actin bundles that extend from the front of the cell allowing for directional movement. Actin-generated forces are important for maintaining cell shape and for cell motility. Pushing forces are generated by actin polymerization, whereas pulling forces are generated by the sliding of actin filaments along the filaments of myosin II [5]. Actin polymerization into filaments is tightly controlled by actin-binding proteins [7].

### 2.2. Podocyte Structure

**Figure 1:**
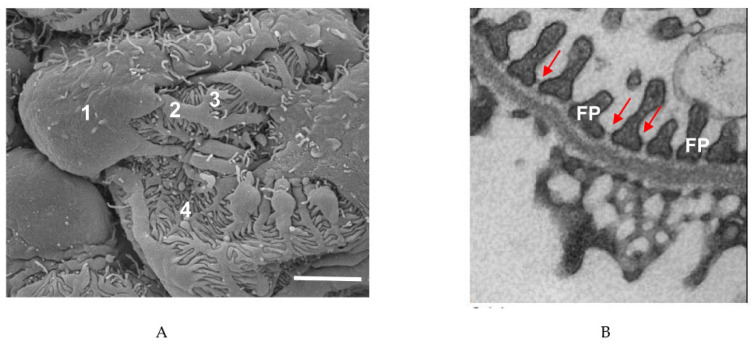
Podocyte structure. (**A**) Scanning electron microscope image of a podocyte. 1, cell body; 2, major process; 3, secondary process; 4, foot process. Scale bar: 3 µm. (**B**) Transmission electron microscope image of podocyte foot processes (FPs) and the slit diaphragms between each process (arrows). Scale bar: 1 µm.

Podocytes, a key part of the glomerular filtration barrier, prevent high-molecular-weight proteins from entering the glomerular ultrafiltrate. These cells have a unique structure which has been preserved over hundreds of millions of years [8]. The podocyte cell body essentially floats in the ultrafiltrate and is attached to the glomerular basement membrane (GBM) only by its processes. Podocyte primary processes divide to form secondary and tertiary processes which ultimately end in membrane extensions called foot processes (FPs), which interdigitate with neighboring podocytes [9,10,11] (Figure 1). Recently, a fourth structural compartment has been identified in podocytes—a ridge-like prominence which extends directly from the basal surface of the cell body and the primary processes [12]. Primary processes contain microtubules whereas foot processes contain actin filaments [13]. In several vertebrate species, FPs contain two types of actin cytoskeleton: a central actin bundle consisting of loosely assembled actin filaments arranged along the FP longitudinal axis surrounded by a cortical actin network consisting of actin filaments bound to cortactin [14]. 

Foot processes can be divided into three domains: the apical membrane domain, the slit diaphragm (SD) and the basal membrane domain which is in contact with the glomerular basement membrane [15]. All three domains are physically linked to the FP actin cytoskeleton. Foot processes of neighboring podocytes are connected by slit diaphragms which are multiprotein complexes that act as signaling hubs. FPs also express large macromolecular complexes called focal adhesions which connect the podocyte cytoskeleton to the extracellular matrix [16]. These adhesions not only allow podocytes to adhere to the basement membrane but also relay signals. As can be seen from the structural organization of the podocyte, the actin cytoskeleton is critical to podocyte structure and function.

FPs do not contain contractile actin fibers yet must adapt to changing tensile and shear stresses. Changes in FP shape are likely mediated by a network of myosin IIA-containing contractile fibers which are found within the podocyte cell body and major processes [17]. It has been proposed that the contractile elements in the cell body and major processes generate tension by exerting force on the non-contractile actin filaments in the FPs. This localized tension on integrins induces the assembly of and strengthens focal adhesion complexes which link integrins to the actin cytoskeleton [17]. These forces are also believed to maintain the space between FPs to allow for efficient filtration. 

During podocyte injury in vivo, foot processes undergo effacement, becoming flatter and wider [18]. Foot process effacement (FPE) is seen in all glomerular diseases and is particularly noticeable in inflammatory diseases which cause rapidly progressive glomerulonephritis [8]. In the first stage of foot process effacement, slit diaphragms disappear or are displaced from their usual place at the base of the foot process and are replaced by occludens-type junctions [8]. In the second and final stage of FPE, the foot processes retract into primary podocyte processes which results in the cell body adhering to the GBM. During this stage, the actin cytoskeleton within the cell body undergoes extensive rearrangement to form a dense “mat” in close proximity to the GBM. In addition, after injury, myosin IIA redistributes from the major processes and cell body to the basolateral aspects of the cell adjacent to the GBM [17]. In animal models of glomerular diseases such as Masugi nephritis, membranous nephropathy and IgA nephropathy, these changes in the cytoskeleton are accompanied by increased expression of actin, α-actinin and synaptopodin [19,20]. Podocyte injury in vitro results in a more motile phenotype which is thought to correspond to FPE in vivo [8,21]. In culture, injured podocytes also develop an increased number of lamellipodia and filopodia. 

**Figure 2:**
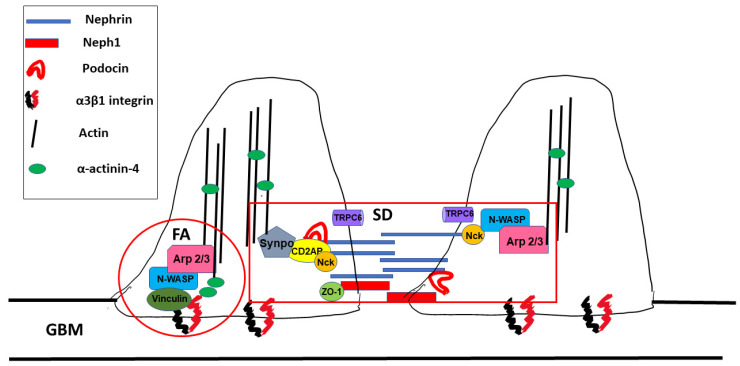
Macromolecular hubs within podocyte foot processes. SD: slit diaphragm, FA: focal adhesion, and GBM: glomerular basement membrane.

Foot processes are dynamic structures and can reassemble after injury as long as the podocyte cell body is attached to the glomerular basement membrane [13]. Actin dynamics in FPs are controlled by two distinct macromolecular protein hubs: the slit diaphragm and focal adhesions (Figure 2).

### 2.3. The Slit Diaphragm

The slit diaphragm (SD) is a size-selective barrier between two podocyte foot processes that prevents filtration of large macromolecules into the ultrafiltrate. The SD is also subject to significant shear stress from glomerular filtrate flow [22] (see Section 3.1 below). The SD contains proteins expressed in adherens and tight junctions such as P-cadherin, zonula occludens-1 (ZO-1) and Fat as well as proteins unique to podocytes alone such as nephrin, nephrin-like 1 (neph1) and podocin [23]. During development, the slit diaphragm (SD) initially appears as a cell–cell junction which then matures into a zipper-like structure which connects adjoining podocytes. The slit diaphragm contains many proteins which are involved in maintenance of the actin cytoskeleton and podocyte structure as well as in signaling. After glomerular injury and as chronic kidney disease progresses, SDs are replaced by tight junctions [24,25]. Conceptually, the slit diaphragm can be thought of as being composed of signal receptors (nephrin, neph1 and podocin) which link to signal integrators or adaptors that then link to the actin cytoskeleton (Figure 2). Below, we describe some of the major components of the SD and their role in podocyte actin cytoskeleton dynamics.

### 2.4. Signal Receptors

#### 2.4.1. Nephrin

Nephrin is a 180 kDa protein encoded by *NPHS1*. Mutations in nephrin result in congenital nephrotic syndrome of the Finnish type [26]. Nephrin is comprised of a cytoplasmic domain, a short transmembrane domain and an extracellular domain with eight IgG-like motifs and a fibronectin type 3 repeat [27]. Nephrin interacts in a homophilic manner with other nephrin molecules and in a heterophilic manner with neph1 proteins via *cis-* and *trans*-interactions (see below) [28,29]. Together, the extracellular domains of nephrin and neph1 form the zipper-like meshwork which creates a sieve-like network with pores with an estimated radius of 12.1 nm each. Nephrin has been shown to be critical for SD formation as without nephrin, the mammalian SD fails to form [30].

#### 2.4.2. Nephrin Signaling

The cytoplasmic portions of both nephrin and neph1 play a major role in signaling and are also important for localizing these proteins to the SD. The cytoplasmic tail of nephrin interacts with podocin (*NPHS2*, see below) which localizes nephrin to lipid rafts within the SD [31]. Extracellular signals are transmitted to the actin core of the each foot process by tyrosine phosphorylation of nephrin. Tyrosine phosphorylation of nephrin occurs in two distinct domains: group A and group B tyrosines [23]. Phosphorylation of either group A or group B tyrosines regulate cytoskeletal organization. Group A tyrosine phosphorylation also modulates cell survival whereas group B mediates nephrin trafficking. In cultured podocytes, tyrosine phosphorylation of nephrin on group A residues mediates lamellipodia formation whereas group B phosphorylation regulates actin polymer extension. Lamellipodia formation in cultured podocytes is regulated through the p85/PI3K/Akt/Cas/Crk pathway [21,32]. Foot process spreading in vivo in response to podocyte injury, a correlate of lamellipodia formation in vitro, is blocked by podocyte-specific knockout of CT 10 regulator of kinase 1/2 (Crk1/2) [21], underscoring the importance of this pathway in FP actin cytoskeletal dynamics. 

Actin extension in podocytes in vitro involves tyrosine phosphorylation and recruitment of non-catalytic region of tyrosine kinase adptor protein (Nck) [33,34]. After nephrin phosphorylation, Nck binds to phospho-nephrin and to neuronal Wiskott–Aldrich syndrome protein (N-WASP) [33,35]. N-WASP then activates the actin-related proteins 2/3 (Arp2/3) complex, a complex of seven proteins including the actin-related proteins Arp2 and Arp3.The Arp2/3 complex is intrinsically inactive because the Arp2 and Arp3 proteins are held apart from each other. Binding of N-WASP to Arp2/3 moves Arp2 and Arp3 closer to each other which promotes de novo actin nucleation and filament branching [36].

In addition to its regulation by tyrosine phosphorylation, the cytoplasmic tail of nephrin also contains a consensus sequence for serine/threonine phosphorylation and can be phosphorylated by protein kinase C alpha (PKCα) [37]. Phosphorylation of nephrin by PKCα results in clathrin and dynamin-mediated endocytosis of nephrin [38], a pathway that is activated in diabetic nephropathy [37,39].

Nephrin signaling is also regulated by tyrosine phosphatases [40]. The Src homology region 2 domain-containing phosphatase-1 (SHP-1) phosphatase which modulates group A tyrosine residues is upregulated in diabetes [41] and another phosphatase, Cl-Ten (Tensin2) modulates podocyte hypertrophy through mammalian target of rapamycin complex 1 (mTORC1) activation [42].

Work from animal models has shown that nephrin tyrosine phosphorylation is required for maintenance of the slit diaphragm. Conversion of all three group B residues in nephrin to phenylalanine which prevents phosphorylation results in mice that rapidly develop foot process effacement and proteinuria after birth [43]. 

Besides regulation through phosphorylation, nephrin also binds the adaptor protein CD2-associated protein (CD2AP, see below). CD2AP can interact directly with actin [44] as well as cortactin [45] and synaptopodin [46] (see below). This complex provides a direct link between the signal receptor nephrin and the FP actin cytoskeleton.

#### 2.4.3. Neph1

The neph1 protein shares some homology with nephrin but contains only five extracellular IgG-like domains [47]. Besides maintaining slit diaphragm integrity through its interactions with nephrin, neph1 also transduce signals from the podocyte exterior. Tyrosine phosphorylation of neph1 results in recruitment of growth factor receptor bound protein 2 (Grb2) and actin polymerization [48,49]. 

#### 2.4.4. Podocin

Podocin is a 383 amino acid protein with a hairpin-like structure with both cytoplasmic N- and C-terminal domains [50,51]. Podocin oligomerizes and is found in lipid rafts, specialized microdomains that are enriched in sphingolipid and cholesterol. Assembly of these lipid rafts clusters nephrin at the SD [51]. Podocin is also required to transport nephrin to the SD [52]. Mutations in *NPHS2*, the gene encoding podocin, are a significant monogenic cause of nephrotic syndrome in children (see below). Besides binding nephrin, the C-terminus of podocin also binds the adaptor protein CD2AP [51]. Podocin also plays a role in formation of tight junctions between neighboring podocytes by associating with and clustering the tight junction proteins coxsackievirus and adenovirus receptor (CAR) and zonula occludens-1 (ZO-1) [53].

### 2.5. Adaptor Proteins/Signal Integrators

#### 2.5.1. Adaptor Proteins

Adaptor proteins play a critical role in linking nephrin and podocin to the actin cytoskeleton of the foot processes [54]. Adaptor proteins are non-catalytic molecules that contain specific domains which allow for protein–protein interactions. Several adaptor proteins have been identified as necessary for maintenance of the FP actin cytoskeleton. CD2AP binds both nephrin and podocin and links these proteins to actin [55]. Knockout of CD2AP in mice results in massive proteinuria and death by 6–7 weeks of age, indicating that CD2AP is necessary for maintenance of the SD [56]. Nck is another adaptor protein that has been extensively studied. Nck binding to nephrin also plays an important role in SD maintenance (see above). Inducible knockout of Nck in mice leads to a reduction in nephrin tyrosine phosphorylation and decreased actin recruitment to the SD [57]. ZO-1 is another adaptor protein that is important for maintenance of SD integrity. ZO-1 interacts directly with neph1 [58] and this interaction is lost when foot processes efface in response to injury [59].

#### 2.5.2. Rho/Small GTPases

The ras homology (Rho) family of small GTPases (RhoA, Ras-related C3 botulinum toxin substrate 1 (Rac1) and cell division control protein 42 homolog (Cdc42)) play a pivotal role in actin dynamics, cell shape and motility. Traditionally, Rac1 and Cdc42 were thought to promote podocyte motility at the leading edge of a migrating cell through formation of lamellipodia (Rac1) and filopodia (Cdc42) whereas RhoA was thought to promote formation of contractile actin and myosin containing stress fibers in the cell body and rear of the cell. In accord with this view, an excess of RhoA activity compared to Rac1/Cdc42 had been shown to result in podocytes with a more stationary phenotype and intact foot processes whereas Rac1 and Cdc42 activation had been shown to promote FP motility and effacement [60,61,62]. However, more recent work has demonstrated that the balance of RhoA to Rac1/Cdc42 activity is central to maintenance of the FP actin cytoskeleton, as both constitutive overexpression and inactivation of RhoA cause FP effacement and proteinuria [63,64]. A variety of factors can modulate RhoA, Rac or Cdc-42 activity in podocytes including calcium influx via transient receptor potential cation channel subfamily C member 6 (TRPC6) [65]. 

#### 2.5.3. Synaptopodin

Synaptopodin is an actin-associated protein expressed in podocyte foot processes [66]. Synaptopodin interacts with α-actinin and this interaction results in bundling and elongation of actin filaments [46]. Synaptopodin also induce stress fibers in cultured podocytes by blocking the Smurf1-mediated ubiquitination and proteasomal degradation of RhoA, resulting in increased RhoA activity [67]. In vitro, synaptopodin suppresses filopodia formation by binding to IRSp53 and blocking the binding of Cdc42 and Mena to insulin receptor substrate p53 (IRSp53) [68]. Treatment of mice with a Mena inhibitor, FP(4)-Mito, reduces proteinuria in a lipopolysaccharide model of podocyte injury [68].

#### 2.5.4. α-actinin-4

α-actinins are a family of proteins that cross-link actin filaments and serve as scaffolds for the assembly of large protein complexes [69]. α-actinin-4 is highly enriched in podocyte foot processes and mutations in this protein are found in FSGS. In addition, dysregulation of α-actinin-4 occurs early in several other forms of nephrotic syndrome including minimal change disease and idiopathic membranous nephropathy [70,71]. This protein has also been shown to be required for podocyte adhesion to the GBM via interactions with integrins [72]. Loss of α-actinin-4 disrupts interactions between the actin cytoskeleton and integrins, decreasing the overall strength of podocyte attachment to the GBM [16].

#### 2.5.5. TRPC6

Transient receptor potential channel 6 (TRPC6) is a non-selective cation channel that is expressed in podocytes at the SD [73,74]. At the SD, TRPC clusters with several other proteins [74,75,76]. Mutations in TRPC6 are associated with FSGS and upregulation of TRPC6 activity has been seen in several proteinuric kidney diseases including autoimmune glomerulonephritis [77]. TRPC6 localization and activity at the SD is modulated by interactions with both nephrin and podocin [78]. In podocytes, TRPC6 also complexes with RhoA [79]. Overexpression of TRPC6 in podocytes results in derangement of the actin cytoskeleton, retraction of podocyte processes, increased cytosolic calcium, activation of RhoA and downregulation of nephrin and synaptopodin [65]. Channel activation in podocytes in vitro or isolated glomeruli has been shown to be mediated by angiotensin II [80,81], diacylglycerol [80], and reactive oxygen species [82]. TRPC6 has also been shown to interact with calpain 1 and 2. The calpains are a family of calcium-dependent cystine proteases that regulate the actin cytoskeleton and cell motility [83]. TRPC6 binding to calpain 1 and 2 induces cleavage of proteins which mediate podocyte adhesion to the GBM, leading to a more motile phenotype. Conversely, knockdown of TRPC6 decreases calpain-initiated cleavage of the podocyte anchoring proteins talin-1, caldesmon-1 and FAK, resulting in podocytes with increased adhesion, decreased motility and increased actin reorganization [83].

### 2.6. Genetic Mutations Affecting Components of the Slit Diaphragm

#### 2.6.1. Mutations Affecting Nephrin and Podocin

Given the critical role that nephrin plays in maintenance of the SD, it is not surprising that mutations affecting the nephrin gene, *NPHS1*, are associated with the severe kidney disease congenital nephrotic syndrome of the Finnish type [26,33,35,84,85,86,87]. Additionally, mutations that alter nephrin trafficking or signaling have also been implicated as causing disease. Examples include mutations in the genes *GTPase activating protein and VPS9 domains 1 (GAPVD1)* and *ankyrin repeat and FYVE domain containing 1 (ANKFY1)* which were identified to cause a recessive type of nephrotic syndrome by impairing nephrin trafficking [88].

Podocin, another crucial protein of the slit diaphragm that interacts with nephrin and CD2AP, has also been implicated in human disease. Mutations in *NPHS2*, which codes for podocin, and *LIM homeobox transcription factor 1 Beta (LMX1B),* a regulator of podocin expression, have been found to cause nephrotic syndrome [89,90,91,92,93]. Since podocin expression is limited to the glomeruli, *NPHS2* mutations typically present without significant extrarenal manifestations [92,93,94]. However *LMX1B* mutations are causative in the syndromic disease nail–patella syndrome characterized by hypoplastic nails and patella, skeletal deformities and varying degrees of nephropathy [89,90,95]. The underlying pathophysiology caused by mutations affecting podocin relates to impaired nephrin signaling due to the inability of nephrin to associate with lipid rafts as well as podocin’s effects on TRPC6 [52,82,96,97,98].

#### 2.6.2. Mutations Affecting Adaptor Proteins/Signal Integrators

CD2AP is scaffolding protein which links nephrin and podocin with the actin cytoskeleton [56,99,100]. Mutations of CD2AP gene have resulted in nephrotic syndrome and FSGS lesions in humans [56,101,102]. The mechanistic cause for disease is still being studied but is believed to be due to CD2AP’s interactions with F-actin through CAPZ as well as its interactions with Rac1 and synaptopodin [15,55,100,103]. Similar to CD2AP, membrane-associated guanylate kinase 2 (MAGI2) is another scaffolding protein that interacts with the nephrin complex and helps maintain the functional structure of the slit diaphragm [104,105]. Mutations in *MAGI2* have been found to cause nephrotic syndrome in mice and humans [106]. MAGI2-associated disease is caused in part due to its interaction with tachykinin 2 (TAC2), which interacts with the Rho GTPase deleted in liver cancer 1 (DLC1), as mentioned above, as well as its interaction with α-actinin-4 and synaptopodin [15,107,108].

As demonstrated in the *LMX1B* mutations causing kidney disease, diseases can arise if transcriptional regulators of slit diaphragm proteins are mutated. MAF BZIP transcription factor B (MAFB) is an important example of this fact as it is a transcription factor that regulates podocin, nephrin, and CD2AP expression [109]. Two mutations in the *MAFB* gene have been identified as causative in two different syndromic diseases where proteinuria is a feature, multicentric carpotarsal osteolysis [110] and focal segmental glomerulosclerosis with Duane retraction syndrome (FSGS-DRS) [111].

Synaptodopodin is intriguing from a disease modulation standpoint since it regulates actin activity through its effects on α-actinin-4 and through RhoA activation and Cdc42 inhibition [67]. Synaptopodin has been shown to have anti-proteinuric effects and its absence has been noted in several different disease states [68,112,113,114]. Furthermore, mutations affecting synaptodopodin expression have been noted in patients with proteinuric kidney disease [115]. A significant amount of research is aimed at examining how to modulate synaptopodin for treatment benefit since synaptopodin has TRPC6 modifying properties and part of the effectiveness of the anti-proteinuric effects of cyclosporin A is due to modification of synaptopodin [113,116]

α-actinin-4 is critical for bundling F-actin but also links integrins that adhere to the basement membrane to the cytoskeleton [16,18]. Mutations affecting the *ACTN4* gene, which codes for α-actinin-4, clinically present as nephrotic syndrome with FSGS but have a wide age range in which disease manifests [115,117,118]. Notably, different mutations in the *ACTN4* genes have demonstrated that the absence of α-actinin-4 as well as mutations causing increased affinity for F-actin, both lead to disease [117,118,119,120]. 

As outlined, regulation of the cytoskeleton dynamic is maintained by a complex balance through RhoA, Rac1, and Cdc42. Given this complexity and need for a balance between all these pathways, numerous gene mutations associated with this system have been identified as causing diseases. Mutations in *Rho GDP dissociation Inhibitor alpha (ARHGDIA*) *and Rho-GTPase activating protein 24 (ARHGAP24)* have both been shown to result in increased Rac1 and Cdc42 activity resulting in disease [121,122,123]. Similarly, mutations in *KN motif and ankyrin repeat domains 1/2/4 (KANK1*, *KANK2,* and *KANK4*) have all been reported to be associated with nephrotic syndrome due to their interactions with proteins that interact with RhoA (KANK 1 interacts with synaptopodin and KANK2 interacts with ARHGDIA) [124]. Mutations in *FAT1*, *intersectin 1* (*ITSN1)*, and *ITSN2* genes have all been reported to cause nephrotic syndrome due to their adverse effects on Cdc42 activation resulting in impaired podocyte migration [107,125]. One of the most studied mutations affecting Cdc42 are the mutations in *inverted formin 2 (INF2*) which codes for a formin protein and has been found to cause Charcot–Marie–Tooth disease as well as FSGS without Charcot–Marie–Tooth disease [126,127,128,129,130,131]. Subsequently, mutations in the *INF2* gene have been found to be one of the most common causes in familial nephrotic syndrome [130,131]. 

DLC1 is another example of a Rho GTPase-activating protein in which mutations cause nephrotic syndrome [107]. Since DLC1 is regulated by cyclic dependent kinase 20 (CDK20) and tensin-2 (TNS2), mutations in *TNS2* and *cyclin-dependent kinase 20 (CDK20)* are also causative in some cases of nephrotic syndrome [107].

Podocalyxin is a cell surface sialomucin expressed by several different cell types including podocytes, vascular endothelium, hematopoietic cells and some subsets of neurons, that is important for podocyte actin cytoskeleton remodeling [132]. Podocalyxin’s C-terminal binding motif (DTHL) interacts with Na^+^/H^+^ exchanger regulator proteins (NHERF1 and NHERF2) and actin-binding protein ezrin, and can modify the cytoskeleton through these interactions [132]. When podocalyxin is knocked down, podocytes develop abnormal cell morphology. Knockout of the *PODXL* gene in mice results in loss of foot processes and loss of the slit diaphragm entirely causing a phenotype of anuric renal failure, omphalocele, and perinatal death [133]. A reported loss-of-function mutation in *PODXL* has been reported clinically with a very similar phenotype to the one observed in PODXL knockout mice [134]. More recently, podocalyxin has been studied outside the kidney for its role in other diseases like cancer, neurologic and atherosclerotic diseases [132,135,136,137].

TPRC6 plays multiple roles in activating RhoA and Rac1 in response to mechanical stress and influences the function of nephrin, podocin, CD2AP and synaptopodin by regulating intracellular calcium [138]. TPRC6 mutations have also been noted in several cases of congenital FSGS [75,139,140,141]. Interestingly, disease severity caused by TRPC6 mutations seems to be dependent on the degree to which they alter intracellular calcium [138,142]. Most of these mutations are gain-of-function mutations mediated in part through angiotensin II that causes excessive intracellular calcium [77,138,139]. There have been mutations reported that result in a loss of function of TRPC6 which cause disease as well [140]. Remarkably, disease presentation can vary widely in age of onset and severity including some families with members with proteinuric disease and some without disease, despite having the same mutation [143]. As such, it has been postulated that a “second hit” is needed to manifest disease. This theory is supported by a transgenic mouse with a TRPC6 mutation that has no evidence of disease at baseline but developed more severe disease after injury than wild type animals [144]. Since TRPC6 is regulated in part by angiotensin II, a significant amount of research is being done to investigate its role in modulating non-congenital glomerular diseases such as diabetic nephropathy and autoimmune glomerulonephritis [138]. Studies looking at modulating TRPC6 in other disease states have yielded some intriguing results [138], but additional studies are needed to determine when and how to optimally alternate TRPC6 signaling in order to derive clinical benefit since both over and under activation of the channel can be associated with disease. 

Phospholipase Cε (PLCε1), a cytoplasmic phospholipase, has been found to be important in podocytes due to its function of regulating internal calcium balance by releasing of calcium from internal stores as well as through activating TRPC6 [65,145]. As mentioned, intracellular calcium affects nephrin and synaptopodin expression in addition to RhoA activation [146,147,148]. Mutations in PLCε1 have been reported to cause SRNS because of its effects on nephrin, synaptopodin, TRPC6 and RhoA [146,149,150].

Glomerular epithelial protien-1 (GLEPP1) is a tyrosine phosphatase expressed on podocyte foot processes and suggested to regulate glomerular pressure and affects nephrin content at the slit diaphragm [151,152]. Decreases in GLEPP-1 have been reported in several diseases including a recessive form of nephrotic kidney disease associated with a mutation on the *GLEPP-1* gene, *PTPRO* [114,151,153,154,155]. Intriguingly, PTPRO knockout mice were reported to be non-proteinuric at baseline but did have significant microscopic abnormalities in podocyte structure and spacing [152].

### 2.7. Focal Adhesions (FAs)

Focal adhesions are specialized complexes within cells where integrin receptors interact with the extracellular matrix outside of the cell and with the actin cytoskeleton within the cell. In podocytes, FAs link the GBM to the actin cytoskeleton of foot processes (Figure 2) and are under significant tensile and shear stress forces. To withstand these forces, the actin cytoskeleton in FAs flows in a retrograde direction in the vicinity of the podocyte membrane in contact with the GBM [13]. In addition, actin is arranged in linear filaments (stress fibers) in the vicinity of FAs and actin filaments are also cross-linked by myosin II and α-actinin, allowing for increased podocyte adhesion to the GBM via actomyosin contractility [13]. Key components of FAs that link the cell exterior to the FP actin array include integrins and GTPases.

#### 2.7.1. Integrins

Integrins link the cytoskeleton to the extracellular matrix and are composed of α and ß subunits. Integrins are classified as laminin-, collagen- or arginine-glycine-aspartic acid-binding receptors based on their heterodimeric composition which allows for binding to different components of the extracellular matrix. In podocytes, the integrin most critical for maintaining the GFB is the laminin-binding integrin α3β1 [156]. In mice, podocyte-specific knockout of either α3 or β1 integrin results in severe proteinuria and death and in humans mutations in the gene encoding α3 result in severe congenital nephrotic syndrome and a highly disorganized GBM [157,158]. 

In cultured podocytes, integrin ligation results in tyrosine phosphorylation of nephrin [159]. Clustering of integrins also leads to activation of focal adhesion kinase (FAK) [160]. FAK plays an important role in cell motility and migration including lamellipodia formation and the dynamic assembly or disassembly of focal adhesion complexes by regulating Rho and Rac activity [161,162,163]. Activation of integrin α3β1 also leads to actin polymerization through activation of the Arp2/3 complex, cortactin and the WASP family of proteins [13]. α3β1 integrins are linked to Arp2/3 via vinculin [13]. Podocyte-specific knockout of vinculin results in podocytes with altered FA size, mislocalization of ZO-1 and increased cell migration [164].

While integrin α3β1 is critical for normal podocyte function, podocyte injury leads to activation of the constitutively inactive podocyte integrin αvβ3. Activation of αvβ3 integrin is thought to form a complex between soluble urokinase-type plasminogen activator receptor (suPAR), apolipoprotein 1 (ApoL1) and α3βv integrin which propagates podocyte detachment from the GBM [165]. suPAR-induced activation of this integrin also increases activity of TRPC6 [13].

Integrins are linked to the actin cytoskeleton by several cytoskeletal adaptor proteins including talin1. In mice, loss of talin1 in podocytes results in significant disruption of the actin cytoskeleton [166]. In murine models of glomerular disease, there is increased calpain-induced cleavage of talin1 which also results in disruption of the actin cytoskeleton [166].

#### 2.7.2. GTPases

At FAs, the adaptor protein vinculin links integrins directly with protein complexes involved in actin polymerization including Arp2/3, cortactin and N-WASP [13]. The Rho-A-associated protein kinase ROCK is also involved in stress fiber and focal adhesion formation. ROCK induces formation of stress fibers and FAs by phosphorylating myosin light chain. This promotes actin binding by myosin II and increases foot process contractility [167]. 

Dynamin is another GTPase which is important for FA formation and function in podocytes. In contrast to Rho, Rac and Cdc42 which are small GTPases, dynamin is a large multidomain protein. Dynamin colocalizes with actin and is involved in growth cone and filopodia formation [168,169,170]. Dynamin can interact directly with actin [171,172] and, after forming dynamin rings, can displace the capping protein gelsolin from the barbed ends of actin filaments promoting actin polymerization [173].

### 2.8. Genetic Mutations Affecting FA Proteins

Since integrin α3β1is the primary integrin adhering the podocyte to the GBM, mutations in genes *ITGA3* and *ITGB1* which code for integrin α3 and β1, respectively, have been reported to cause a syndromic disease in humans of nephrotic syndrome, interstitial lung disease, and epidermolysis bullosa [158,174]. Similarly, since CD151 is important for proper insertion of integrin α3β1, mutations in the gene *CD151* cause a similar syndromic disease of nephropathy, pretibial epidermolysis bullosa and deafness [175]. 

Epithelial membrane protein 2 (EMP2) is an interesting protein in that it has been found to perform several functions including cell adhesion, trafficking of GPI proteins into lipid rafts, organization of caveolar regulation, and is involved in vascular endothelial growth factor A- (VEGFA-) mediated angiogenesis [176]. Mutations in the *EMP2* gene have been proposed to cause a congenital nephrotic syndrome that was supported by abnormalities noted in cultured human podocytes and zebrafish models with the mutation [177,178]. Interestingly, global and podocyte-specific EMP2 knockout mice do not have proteinuria or abnormal glomerular histology [176]. Given the conflicting animal data and limited number of nephrotic patients with this gene mutated, further research is needed. Disorders of *EMP2* may ultimately represent genetic mutations that requires a “second hit” injury before the development of disease.

Remarkably, gene mutations in the integrin linker proteins (paxillin and talin), signaling kinases *focal adhesion kinase* (*FAK)*, *integrin-linked protein kinase (ILK)*, complex protein *PINCH*, and kindlin-2 have not been reported to be causative in human kidney disease. This might be explained by the fact that global knockouts in these genes in mice leads to significant embryonic abnormalities that lead to non-viable embryos, supporting the critical nature of these proteins [179,180,181,182,183,184]. 

### 2.9. Other Actin Associated Genetic Mutations

Actin by itself is inert and needs appropriate bundling and coupling with myosin to form the contractile apparatus that provides the mechanical force needed for movement. Since actin needs myosin for movement, it is not surprising to learn that mutations affecting myosin can cause diseases which alter the actin cytoskeleton. Most notable in regards to podocytes are mutations in the *MYH9* gene, which encodes non-muscle myosin class II isoform A, and *MYO1E*, which is a membrane-associated class I myosin [185,186]. Mutations in *MYH9* are associated with an autosomal dominant pattern of familial nephritis, deafness, and macrothrombocytopenia but has been reported to have significant variation in the degree of renal disease [187]. On the other hand, *MYO1E* is a recessive mutation associated with FSGS without extrarenal manifestations, likely due to the specialized location and the role MYO1E plays in the podocyte [185].

Without appropriate actin binding, actin and myosin dynamics are impaired, leading to ineffective contractions. Proteins anillin, coded on the *ANLN* gene, and advillin, coded on *AVIL* gene, are both F-actin-binding proteins needed for podocyte motility and both have mutations that are associated with kidney disease [188,189]. Outside of their F-actin-binding function, anillin and advillin also interact with CD2AP and PLCε1, respectively, of the slit diaphragm [188], to regulate and coordinate motility while maintaining the filtration barrier [147,189]. 

## 3. Other Mediators of Podocyte Actin Dynamics

While structural components of the SD or FAs are the best-studied modulators of actin cytoskeleton dynamics in podocytes, other factors have also been shown to alter the actin cytoskeleton in podocytes. These include hemodynamic and shear forces, cytokines, hormones and drugs.

### 3.1. Hemodynamic Factors

As mentioned above, podocyte foot processes are exposed to both tensile and shear stress [190]. The glomerular filtrate has the highest extravascular fluid flow anywhere in the body in terms of both volume and velocity [22,191]. Conditions such as hypertension and the hyperfiltration seen in early diabetic nephropathy, the solitary kidney or secondary focal segmental glomerulosclerosis result in significant increases in the hemodynamic stresses on podocyte FPs, leading to podocyte detachment from the GBM [2,192,193]. Since podocytes are terminally differentiated cells, detachment of significant numbers of podocytes from the GBM and loss of these podocytes in the urine leads to irreversible progression of kidney disease [194]. Thus, maintaining podocyte structural integrity is critical to preserving renal function. Increases in glomerular hemodynamic forces initiate a series of events within FPs starting with replacing slit diaphragms with occluding junctions, and proceeding to retraction of processes leading to direct contact between the podocyte cell body and the GBM [195,196].

In vitro, podocytes respond to shear stress by reorganizing the actin cytoskeleton resulting in loss of stress fibers, loss of vinculin from focal adhesions and recruitment of α-actinin to sites of cell–cell contact [197]. Increased shear stress also increases podocyte motility in vitro leading to membrane ruffling and lamellipodia formation. 

### 3.2. Cytokines/Hormones

#### 3.2.1. TGF-ß1

Expression of transforming growth factor beta 1 (TGF-β1) is upregulated in diabetic nephropathy [198] and in cultured podocytes exposed to high glucose [199]. Treatment of cultured podocytes with TGF-β1 results in shortening of podocyte foot processes and the formation of broad tight junctions between podocytes as well as reduction in the expression of nephrin and ZO-1 [200].

#### 3.2.2. IL-6

Podocytes express interleukin-6 (IL-6) as well as the IL-6 receptor and can secrete and directly respond to IL-6 in response to proinflammatory stimuli [201,202,203]. Treatment of cultured podocytes with IL-6 has been shown to increase podocyte motility via signal transducer and activator of transcription (STAT3)-induced phosphorylation of myosin light chain, leading to decreased stress fiber formation and decreased focal adhesion size [204]. 

In podocytes, knockout of a trafficking protein, the neonatal Fc receptor (FcRn) which sorts both monomeric IgG and immune complexes, significantly reduces IL-6 production after an immune challenge. FcRn KO podocytes are also hypermotile at baseline and have significantly shorter stress fibers. These defects can be rescued by treatment with IL-6 [205]. FcRn-mediated modulation of the actin cytoskeleton via IL-6 provides further evidence that protein trafficking in podocytes is linked to actin cytoskeletal dynamics.

#### 3.2.3. Angiotensin II (Ang II)

In cultured podocytes, AngII activates Rac-1, increases lamellipodia formation and leads to a more motile podocyte phenotype [206]. Ang II also induces phosphorylation of the actin-binding protein complex ezrin/radixin/moesin leading to F-actin reorganization. In vivo, Ang II increases nephrin binding to ß-arrestin 2, leading to nephrin endocytosis and increased glomerular permeability [207].

### 3.3. Drugs

#### 3.3.1. Renin-Angiotensin System (RAS) Inhibitors

RAS inhibition decreases hemodynamic stress on podocytes by reducing intraglomerular pressure [208]. RAS inhibition has also been shown preserve podoocyte structure independent of pressure related effects. In Munich Wistar Froemters rats which develop proteinuria spontaneously with age, treatment with isinopril prevents redistribution of ZO-1 [209]. In addition, the angiotensin receptor blocker valsartan attenuates the decrease in nephrin seen in rats with diabetic nephropathy [210].

#### 3.3.2. Glucocorticoids (GCs)

Podocytes express glucocorticoid receptors [211] and GCs have been shown to have a direct effect on the podocyte actin cytoskeleton by upregulating nephrin [212]. In addition, treatment of cultured podocytes with dexamethasone increases the stability of the actin cytoskeleton in podocytes by increasing the total cellular quantity of polymerized actin and increasing RhoA activity [213].

#### 3.3.3. Cyclosporin A (CsA)

Cyclosporin inhibits the serine/threonine phosphatase calcineurin and is used as a treatment for various proteinuric kidney diseases including FSGS and membranous nephropathy. While CsA is an immune modulator, it has also been shown to block the calcineurin-mediated dephopshorylation of synaptopodin, protecting synaptopodin from degradation by cathepsin-L. Since synaptopodin regulates actin dynamics through modulating RhoA and cdc42 (see above), CsA may exert a direct effect on the actin cytoskeleton in podocytes.

#### 3.3.4. Rituximab

Rituximab is another immunosuppressive medication that may also directly modulate the actin cytoskeleton in podocytes. Rituximab has been shown to partially prevent sphingomyelin phosphodiesterase acid-like 3b (SMPDL3b) expression in podocytes and actin cytoskeleton dynamics in podocytes are affected by SMPDL3b levels [214,215].

#### 3.3.5. Abatacept

Abatacept is a B7-1 inhibitor used in a variety of rheumatologic diseases and after kidney transplant as an immune modulator. B7-1 is expressed in murine podocytes and upregulated after podocyte injury [216]. Mice lacking B7-1 are protected against lipopolysaccharide-induced proteinuria [216]. In a small study of patients with primary FSGS and FSGS after kidney transplantation, abatacept was shown to decrease podocyte migration in response to injury, restore β1 integrin levels and decrease proteinuria [217].

#### 3.3.6. Dasatanib

Dasatanib is a tyrosine kinase inhibitor used to treat chronic myelogenous leukemia and acute lymphoblastic leukemia. Dasatanib directly disrupts the actin cytoskeleton in cultured podocytes and reduces the number of focal adhesions [218]. Treatment of podocytes with dasatanib also significantly downregulates expression of multiple proteins associated with actin cytoskeleton regulation. In vivo, administration of dasatanib to mice results in foot process effacement and decreased synaptopodin expression [218].

## 4. Clinical Presentation of Genetic Mutations in Podocyte Actin

### Clinical Presentation

Clinically, patients with disorders of the podocyte actin cytoskeleton present with proteinuria, edema, and hypoalbuminemia consistent with nephrotic syndrome. Histologic evaluation of kidney biopsies in these patients often shows a focal segmental glomerulosclerosis (FSGS) pattern of glomerular injury. Patients with proteinuric kidney diseases are subgrouped based on their lack of response to glucocorticoid therapy (steroid sensitive nephrotic syndrome (SSNS) or steroid resistant nephrotic syndrome (SRNS)) [125]. Since 1998, when mutations within the gene *NPHS1* were identified to cause congenital nephrotic syndrome of the Finnish type [26], SRNS has been largely attributed to genetic mutations that lead to impaired function of the glomerular filtration barrier. Newer evidence suggests that genetic mutations may not only cause SRNS but may be associated with some causes of SSNS since they are at least partially responsive to glucocorticoid treatment [107]. This would suggest that nephrotic syndrome should be classified by means other than clinical responsiveness to treatment.

Though the inciting cause for the disease may be diverse, the insult subsequently causes cytoskeleton remodeling that results in foot process effacement, primary foot process retraction, loss of the subpodocyte space, and formation of dense microfilaments linking close to the GBM [2,8,16,219]. This initial response is felt to be adaptive, as it results in the podocytes forming more secure attachments to the basement membrane in order to prevent cell detachment [191,220]. If the disease state persists, podocytes can detach which results in a cascading event resulting in other podocytes detaching [219]. As podocytes detach, parietal epithelial cells from Bowman’s capsule migrate to the exposed basement membrane, as a reparative mechanism [221,222]. The capsular adhesions to the basement membrane and sclerosis that are characteristic of FSGS are caused by the parietal epithelium being exposed to the cytokines and chemokines released by the detaching podocytes, in addition to increased mechanical stress [219,223].

Over 70 genes have been identified which have mutations that can result in proteinuria and nephrotic syndrome [224]. The number of genes associated with nephrotic syndrome is likely to continue to grow as technology advances since current methodology still has not identified a causative mutation in a large portion of patients with SRNS [225,226]. This has given rise to the term phenocopy, which is when a patient has the signs and symptoms consistent with a monogenic mutation (phenotype) but lack the genetic mutations (genotype) known to cause that disease [224,225,227]. Table 1 contains a list of genes associated with nephrotic syndrome that are a result of abnormalities within the cytoskeleton or due to abnormal protein signaling causing aberrant cytoskeleton function. 

## 5. Conclusions

The actin cytoskeleton in podocyte foot processes is essential for normal podocyte structure and function. Regulation of actin cytoskeleton dynamics in FPs is a complex and tightly regulated process. Perturbations in actin dynamics in podocytes, which can be due to acquired or genetic factors, leads to foot process effacement, proteinuria and often progression of kidney disease. An understanding of actin regulation in podocytes will lead to targeted therapies to treat proteinuric kidney diseases.

## Figures and Tables

**Table 1:** Genes associated with mutations causing disease.

Gene	Protein	Function
Focal Adhesions		
*CD151*	CD151	Transmembrane protein regulator
*EMP2*	Epithelial membrane protein 2	Cell adhesion and trafficking protein
*ITGA3*	Integrin α3	Anchoring protein
*ITGB1*	Integrin β1	Anchoring protein
Slit Diaphragm		
*ANKFY1*	Rabakyrin-5	Nephrin trafficking
*CD2AP*	CD2-associated protein	Slit diaphragm linking protein to actin cytoskeleton
*GAPVD1*	GTPase-activating protein and VPS9 domain-containing protein 1	Nephrin trafficking
*LMX1B*	LIM homeobox transcription factor 1-β	Transcription regulator of podocin
*MAFB*	Transcription factor MafB	Transcription regulator of nephrin, podocin, CD2AP
*MAGI2*	Membrane-associated guanylate kinase	Scaffolding protein for nephrin complex
*NPHS1*	Nephrin	Slit diaphragm signaling protein
*NPHS2*	Podocin	Mechanosensing protein linking plasma membrane to actin cytoskeleton
*PLCε1*	Phospholipase Cε1	Slit diaphragm signaling protein
*TPRC6*	Transient receptor potential channel 6	Regulates Ca^2+^ signaling for mechanosensation Activates RhoA and Rac1
Motility/Actin Dynamics		
*ACTN4*	α-actinin-4	Links focal adhesions to actin cytoskeleton
*ANLN*	Anillin	Scaffold protein linking RhoA with actin
*ARHGDIA*	Rho GDP-dissociation inhibitor α	Regulates RhoGTPase signaling
*ARHGAP24*	Rho GTPase-activating protein 24	Regulates RhoGTPase signaling
*AVIL*	Advillin	Ca^2+^ regulated actin-binding protein
*CDK20*	Cyclin-dependent kinase 20	Regulates RhoA/Rac through regulating DLC1
*DLC1*	Rho GTPase-activating protein 7	Regulates RhoGTPase signaling
*INF2*	Inverted formin 2	Cuts actin filaments
*ITSN1* *ITSN2*	Guanin exchange factor proteins	Activates Cdc42
*FAT1*	Fat cadherin 1	Connects slit diaphragm and actin cytoskeleton
*KANK1* *KANK2* *KANK4*	Kidney ankyrin repeat-containing protein	Regulates actin polymerization
*MAGI2*	Membrane-associated guanylate kinase	Scaffold protein
*MYH9*	Heavy chain of non-muscle myosinIIA	Contractile protein
*PODXL*	Podocalyxin	Modulates actin cytoskeleton
*PTPRO*	Glomerular epithelial protein 1 (GLEPP1)	Glomerular pressure maintenance
*SYNPO*	Synaptopodin	Actin-associated protein for foot process motility
*TNS2*	Tensin-2	Motility regulating through MAGi2 interaction
